# Supplementary material for: Adjuvant Effect of Molecular Iodine in Conventional Chemotherapy for Breast Cancer. Randomized Pilot Study
Source: Nutrients. 2019 Jul 17;11(7):1623. doi: 10.3390/nu11071623 (PMC6682905; doi:10.3390/nu11071623)
Supplement: Supplementary file 1 [file nutrients-11-01623-s001.pdf]

Figure S1

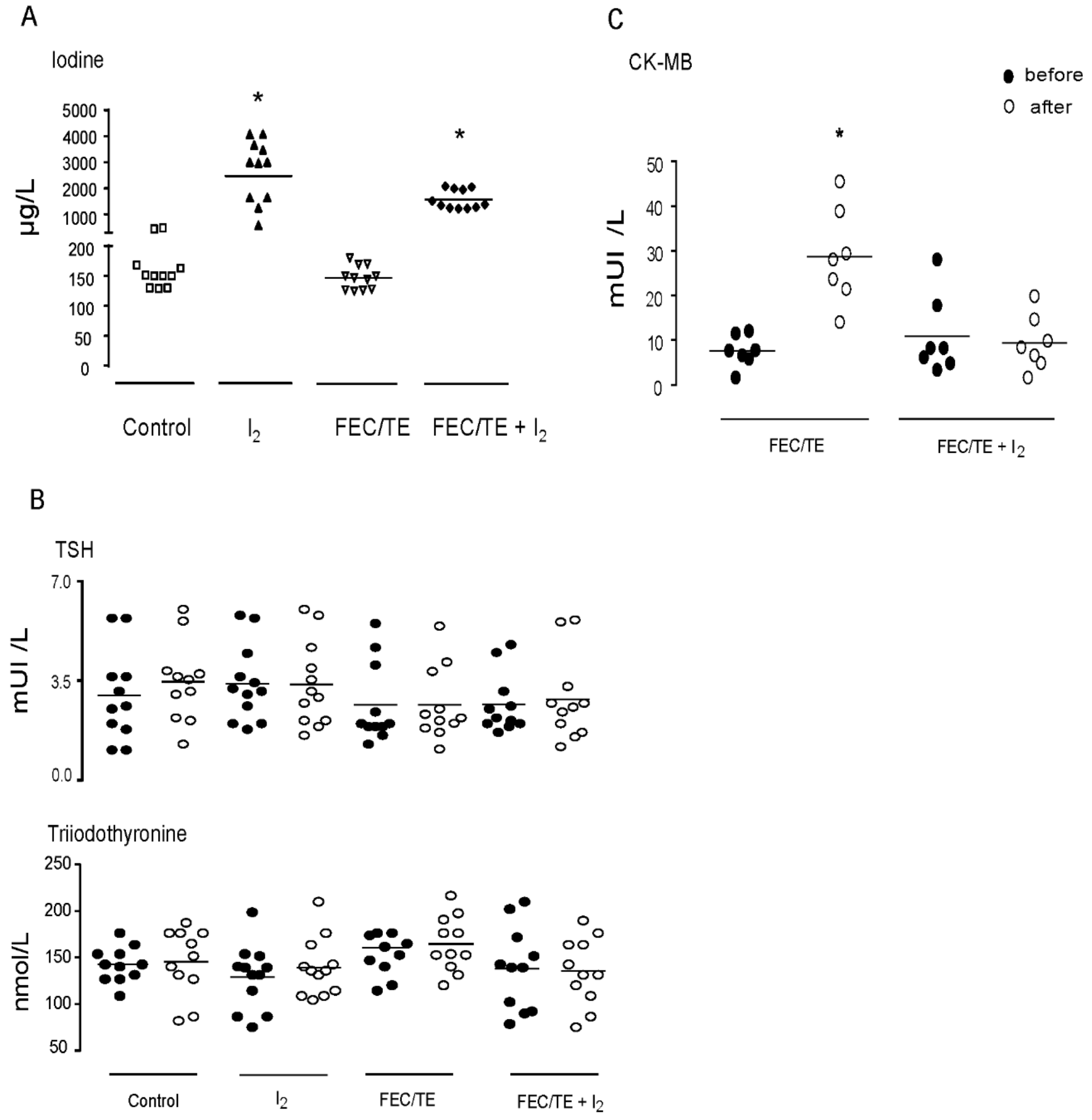
A
Iodine
µg/L
5000
4000
3000
2000
1000
200
150
100
50
0
*
*
Control
$I_2$
FEC/TE
FEC/TE + $I_2$
C
CK-MB
before
after
mUI /L
50
40
30
20
10
0
*
FEC/TE
FEC/TE + $I_2$
B
TSH
mUI /L
7.0
3.5
0.0
Triiodothyronine
nmol/L
250
200
150
100
50
Control
$I_2$
FEC/TE
FEC/TE + $I_2$

Figure S2

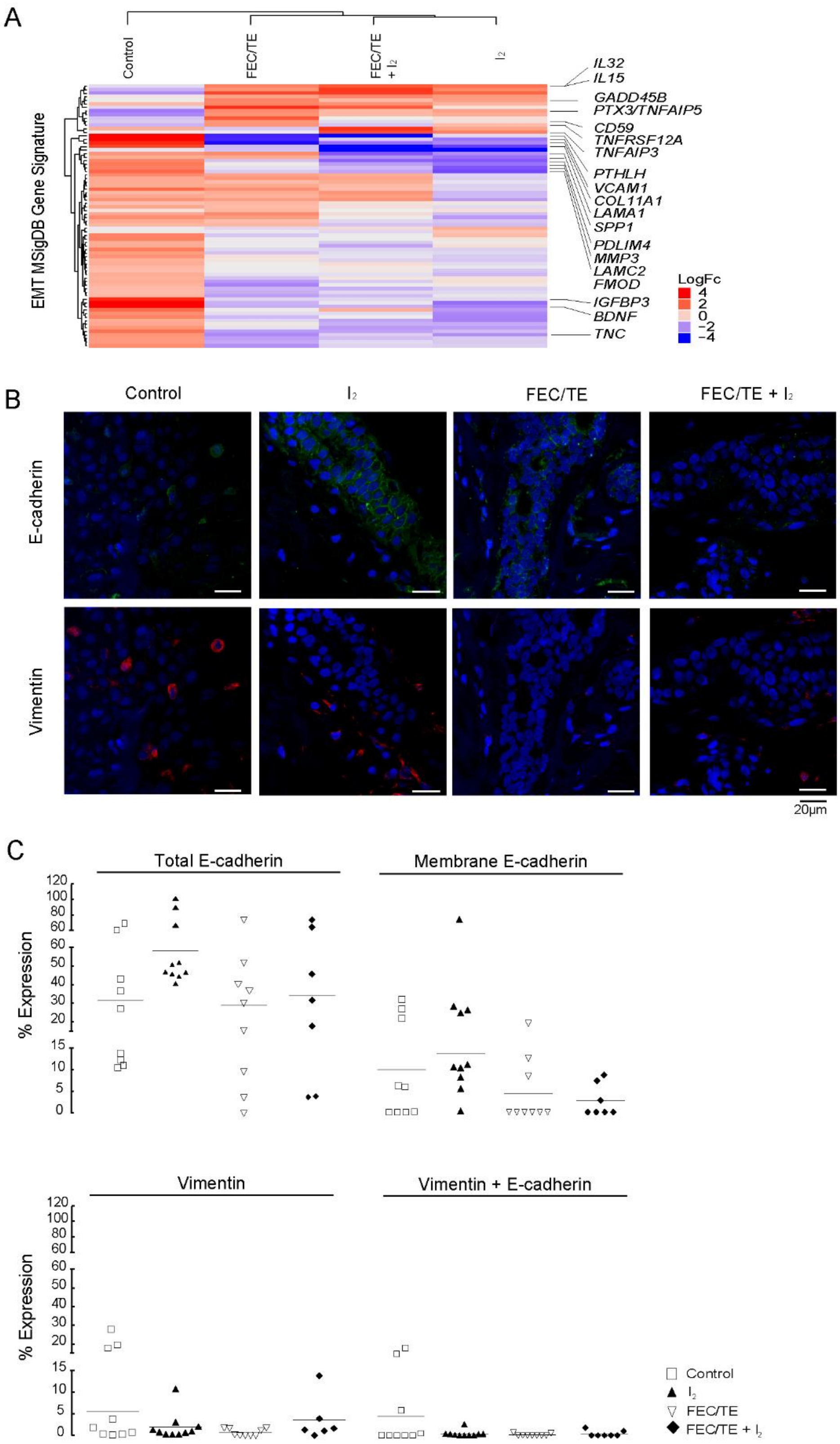

A
Control
FEC/TE
FEC/TE + I2
I2
EMT MSigDB Gene Signature
IL32
IL15
GADD45B
PTX3/TNFAIP5
CD59
TNFRSF12A
TNFAIP3
PTHLH
VCAM1
COL11A1
LAMA1
SPP1
PDLIM4
MMP3
LAMC2
FMOD
IGFBP3
BDNF
TNC
LogFc
4
2
0
−2
−4
B
Control
I2
FEC/TE
FEC/TE + I2
E-cadherin
Vimentin
20µm
C
Total E-cadherin
Membrane E-cadherin
Vimentin
Vimentin + E-cadherin
% Expression
Control
I2
FEC/TE
FEC/TE + I2

Table S1. Oligonucleotides used for RT-qPCR

| Gene | Reference | Sense | Antisense | bp |
|---|---|---|---|---|
| *CDKN1A* | NM_001291549.1 | GACCATGTGACCTGTCACT | GCGGATTAGGGCTTCCTCTT | 176 |
| *BAX* | NM_138764.4 | AAGCTGAGCGAGTGTCTCAAGCGC | TCCCGCCACAAAGATGGTCACG | 327 |
| *BIRC5* | NM_001012271.1 | TTCTCAAGGACCACCGCATC | CCAAGTCTGGCTCGTTCTCA | 126 |
| *BCL2* | NM_000633.2 | GTGGAGGAGCTCTTCAGGGA | AGGCACCCAGGGTGATGCAA | 306 |
| *HIF1A* | NM_001243084.1 | TTGATGGGATATGAGCCAGA | TGTCCTGTGGTGACTTGTCC | 128 |
| *CTB* | NM_001101.3 | CCATCATGAAGTGTGACGTTG | ACAGAGTACTTGCGCTCAGGA | 175 |

Table S2. Early breast cancer patients- Clinical information

| Patient | age | Classification | grade | Estrogen receptor biopsy/tumor | Treatment | Days treatment | Treatment completed |
|---|---|---|---|---|---|---|---|
| 1 | 42 | Ductal | II | +/+ | P | 19 | yes |
| 2 | 40 | Ductal | II | +/+ | P | 21 | yes |
| 3 | 43 | Ductal | II | +/+ | P | 16 | yes |
| 4 | 44 | Ductal | II | +/ | $I_2$ | 15 | no |
| 5 | 49 | Ductal | II | +/+ | $I_2$ | 16 | yes |
| 6 | 49 | Ductal | II | +/+ | P | 14 | yes |
| 7 | 49 | Ductal | II | +/+ | $I_2$ | 16 | yes |
| 8 | 61 | Ductal | II | +/+ | $I_2$ | 7 | yes |
| 9 | 38 | Ductal | II | +/+ | $I_2$ | 23 | yes |
| 10 | 81 | Ductal | II | +/+ | $I_2$ | 35 | yes |
| 11 | 45 | Ductal | II | +/+ | $I_2$ | 21 | yes |
| 12 | 51 | Ductal | II | +/+ | P | 12 | yes |
| 13 | 51 | Ductal | II | +/+ | P | 21 | yes |
| 14 | 41 | Ductal | II | +/+ | P | 21 | yes |
| 15 | 71 | Ductal | II | +/+ | P | 41 | yes |
| 16 | 90 | Ductal | II | +/+ | P | 12 | yes |
| 17 | 47 | Medullar | II | +/+ | P | 17 | yes |
| 18 | 53 | Ductal | II | +/+ | $I_2$ | 17 | yes |
| 19 | 44 | Ductal | II | +/+ | $I_2$ | 27 | yes |
| 20 | 46 | Medullar | II | +/+ | $I_2$ | 29 | yes |
| 21 | 47 | Ductal | II | +/+ | $I_2$ | 10 | yes |
| 22 | 78 | Ductal | II | +/+ | $I_2$ | 11 | yes |
| 23 | 78 | Ductal | II | +/+ | $I_2$ | 15 | yes |
| 24 | 59 | Lobular | II | +/+ | P | 13 | yes |
| 25 | 65 | Lobular | II | +/ | P | 18 | no |
| 26 | 64 | Lobular | II | +/ | $I_2$ | 9 | no |
| 27 | 59 | Ductal | II | +/ | P | 10 | no |
| 28 | 61 | Ductal | II | +/ | P | 15 | no |
| 29 | 62 | Ductal | II | +/ | $I_2$ | 8 | no |
| 30 | 58 | Ductal | II | +/ | $I_2$ | 7 | no |

Table S3. Late breast cancer patients-Clinical information

| Patient | age | Classification | grade | Estrogen receptor biopsy/tumor | Therapy cycles | Treatment | Treatment completed |
|---|---|---|---|---|---|---|---|
| 51 | 36 | Ductal | IIIA | +/+ | FEC 4 | $I_2$ | yes |
| 52 | 45 | Ductal | IIIA | -/+ | FEC 6 | $I_2$ | yes |
| 53 | 57 | Ductal | IIIB | -/+ | FEC 4 | $I_2$ | yes |
| 54 | 43 | Ductal | IIIA | -/+ | FEC 4 | $I_2$ | yes |
| 55 | 60 | Ductal | IIIA | -/- | FEC 4 | P | yes |
| 56 | 38 | Ductal | IIIA | -/- | FEC 4 | P | yes |
| 57 | 67 | Ductal | IIIB | -/+ | FEC 4 | P | yes |
| 58 | 49 | Ductal | IIIA | +/+ | TE 6 | $I_2$ | yes |
| 59 | 36 | Ductal | IIIB | -/- | FEC 4 | P | yes |
| 60 | 41 | Ductal | IIIB | +/+ | TE 6 | $I_2$ | yes |
| 61 | 34 | Ductal | IIIA | +/+ | TE 6 | P | yes |
| 62 | 55 | Lobular | IIIB | +/+ | FEC 4 | $I_2$ | yes |
| 63 | 46 | Lobular | IIIA | -/+ | FEC 4 | $I_2$ | yes |
| 64 | 43 | Lobular | IIA | +/+ | FEC 4 | P | yes |
| 65 | 54 | Ductal | IIIA | +/+ | FEC 4 | $I_2$ | yes |
| 66 | 52 | Ductal | IIIB | +/+ | FEC 4 | P | yes |
| 67 | 40 | Ductal | IIIA | +/+ | FEC 4 | $I_2$ | yes |
| 68 | 42 | Ductal | IIIA | -/- | FEC 4 | P | yes |
| 69 | 27 | Lobular | IIIB | -/- | TE 6 | $I_2$ | yes |
| 70 | 61 | Lobular | IIIB | +/ | FEC 4 | P | no |
| 71 | 44 | Ductal | IIIB | +/+ | FEC 4 | P | yes |
| 72 | 43 | Ductal | IIIB | +/+ | FEC 4 | P | yes |
| 73 | 41 | Ductal | IIIB | +/ | FEC 4 | P | no |
| 74 | 36 | Ductal | IIIIB | +/ | FEC 4 | P | no |
| 75 | 33 | Ductal | IIIB | +/ | FEC 4 | P | no |
| 76 | 29 | Ductal | IIIB | +/ | FEC 6 | P | no |
| 77 | 44 | Ductal | IIIA | +/ | FEC 4 | P | no |
| 78 | 49 | Lobular | IIIA | +/+ | FEC 4 | P | yes |
| 79 | 50 | Ductal | IIIB | +/ | FEC 4 | P | no |
| 80 | 56 | Ductal | IIIB | +/ | FEC 4 | P | no |

Table S4. Expression change of genes involved in Th1 and Th2 differentiation pathways in the different treatment groups.

| | | Control | | $I_2$ | | FEC/TE | | FEC/TE $+I_2$ | |
|---|---|---|---|---|---|---|---|---|---|
| **T-Cell** | **Gene Symbol** | LogFC | FDR | LogFC | FDR | LogFC | FDR | LogFC | FDR |
| Th1 | **TBX21** | **-1.24** | 2.01E-06 | **2.31** | 7.01E-33 | **1.47** | 3.86E-08 | **2.70** | 3.19E-42 |
| Th1 | **IL12RB1** | **-1.38** | 6.84E-11 | **2.18** | 4.61E-38 | 0.25 | *ns* | **3.11** | 7.37E-89 |
| Th1 | **IL12RB2** | -0.73 | 2.18E-02 | **3.67** | 2.05E-86 | -0.62 | *ns* | **1.91** | 2.17E-16 |
| Th1 | **IL27RA** | *-0.03* | *ns* | **1.48** | 5.42E-42 | 0.11 | *ns* | **1.20** | 4.22E-26 |
| Th1 | **GP130** | 0.72 | 1.02E-03 | 0.56 | 1.56E-05 | 0.11 | *ns* | **1.33** | 1.51E-27 |
| Th1 | **STAT1** | -0.93 | 5.72E-11 | **1.73** | 6.47E-75 | -0.24 | *ns* | **1.36** | 2.47E-46 |
| Th1 | **STAT4** | **-1.19** | 1.69E-07 | **2.19** | 1.79E-36 | **1.56** | 1.29E-10 | **3.52** | 3.59E-104 |
| Th1 | **JAK2** | -0.62 | 9.50E-03 | **1.40** | 2.94E-28 | 0.03 | *ns* | **2.16** | 1.17E-67 |
| Th1 | **IRF1** | -0.96 | 1.26E-10 | **1.64** | 1.75E-60 | 0.51 | 4.10E-04 | **1.78** | 6.08E-69 |
| Th1 | **IL18** | **-1.23** | 5.23E-11 | **1.74** | 2.24E-33 | **1.38** | 1.73E-15 | **2.04** | 5.89E-51 |
| Th1 | **IL18RAP** | **-1.45** | 3.76E-06 | **2.96** | 6.04E-41 | **1.61** | 6.13E-05 | **3.26** | 1.44E-46 |
| Th1 | **IFNGR1** | 0.42 | 7.06E-03 | 0.54 | 3.63E-07 | -0.14 | *ns* | 0.45 | 1.52E-05 |
| Th1 | **IFNGR2** | *-0.10* | *ns* | -0.28 | 1.37E-02 | 0.62 | 2.16E-05 | 0.06 | *ns* |
| Th1 | **TNFA** | **-1.69** | 3.33E-13 | **1.97** | 1.60E-24 | **1.01** | 2.22E-04 | **3.05** | 3.37E-62 |
| Th1 | **TNFB** | **-2.28** | 2.48E-11 | **2.28** | 2.52E-13 | **1.86** | 1.78E-05 | **4.49** | 7.11E-77 |
| Th2 | **GATA3** | **3.23** | 3.08E-97 | **-2.45** | 5.10E-133 | **-1.08** | 1.16E-12 | **-1.20** | 9.88E-34 |
| Th2 | **TSLP** | **1.74** | 4.91E-06 | **-2.46** | 3.41E-10 | **-1.69** | 1.36E-04 | **-1.12** | 2.27E-04 |
| Th2 | **DEC2** | **1.26** | 2.35E-14 | **-1.03** | 1.35E-19 | -0.12 | *ns* | -0.23 | 4.92E-02 |
| Th2 | **IL13RA1** | 0.77 | 5.07E-06 | 0.05 | 6.83E-01 | 0.09 | *ns* | -0.28 | 6.97E-03 |
| Th2 | **AREG** | 0.67 | *ns* | **-2.74** | 2.79E-08 | -0.36 | *ns* | **-1.25** | 9.40E-04 |
| Th2 | **IL4R** | 0.44 | 7.86E-03 | -0.08 | 5.26E-01 | 0.15 | *ns* | 0.94 | 2.40E-18 |
| Th2 | **IL33** | 0.02 | *ns* | -0.24 | 1.25E-01 | 0.42 | 1.51E-02 | **1.94** | 1.45E-68 |
| Th2 | **STAT5A** | -0.07 | *ns* | 0.38 | 3.11E-04 | 0.91 | 1.71E-10 | 0.33 | 1.94E-03 |
| Th2 | **CCL22** | -0.46 | *ns* | 0.60 | 9.48E-03 | **3.42** | 4.16E-49 | **2.40** | 3.18E-39 |
| Th2 | **STAT6** | -0.51 | 7.62E-04 | 0.21 | 4.76E-02 | 0.04 | *ns* | 0.71 | 1.76E-12 |
| Th2 | **CCL11** | -0.71 | *ns* | 0.81 | 1.80E-02 | 0.83 | 3.82E-02 | **2.10** | 9.32E-13 |
| Th2 | **FOXP3** | **-1.45** | 4.76E-11 | **1.80** | 9.02E-25 | -0.03 | *ns* | **2.61** | 5.05E-54 |

*ns* : not significant

Table S5. PPARγ positively correlated genes in breast cancer- GO BP enrichment of differentially expressed genes in treatment groups

| | Treatment | GO ID | GO Biological Process | FDR B&H | Genes from input | Total up/down regulated genes per treatment |
|---|---|---|---|---|---|---|
| Upregulated genes logFC>0.58;FDR<0.05 | **Control** | GO:1901700 | response to oxygen-containing compound | 2.661E-3 | 15 | 49 |
| | | GO:0010243 | response to organonitrogen compound | 2.661E-3 | 11 | |
| | | GO:0042127 | regulation of cell proliferation | 7.174E-3 | 14 | |
| | **$I_2$** | GO:0006955 | immune response | 3.261E-4 | 14 | 36 |
| | | GO:0001819 | positive regulation of cytokine production | 4.397E-4 | 8 | |
| | | GO:0045087 | innate immune response | 5.771E-4 | 10 | |
| | **FEC/TE +$I_2$** | GO:1901700 | response to oxygen-containing compound | 5.149E-6 | 26 | 87 |
| | | GO:0009725 | response to hormone | 1.708E-5 | 20 | |
| | | GO:0006955 | immune response | 7.810E-5 | 23 | |
| | **FEC/TE** | GO:1901700 | response to oxygen-containing compound | 8.777E-6 | 30 | 108 |
| | | GO:0009725 | response to hormone | 1.213E-5 | 23 | |
| Downregulated genes logFC<0.58;FDR<0.05 | **Control** | GO:0007155 | cell adhesion | 2.271E-5 | 96 | 96 |
| | | GO:0006955 | immune response | 1.003E-3 | 22 | |
| | | GO:0002684 | positive regulation of immune system process | 9.635E-4 | 17 | |
| | **$I_2$** | GO:0010243 | response to organonitrogen compound | 1.099E-5 | 22 | 109 |
| | | GO:1901700 | response to oxygen-containing compound | 7.586E-5 | 28 | |
| | | GO:0040011 | locomotion | 7.586E-5 | 29 | |
| | | GO:0007155 | cell adhesion | 1.750E-4 | 26 | |
| | **FEC/TE +$I_2$** | GO:0040011 | locomotion | 4.309E-2 | 15 | 58 |
| | | GO:0030198 | extracellular matrix organization | 4.309E-2 | 7 | |
| | **FEC/TE** | ns | ns | ns | ns | 37 |
